# CD44/Folate Dual Targeting Receptor Reductive Response PLGA-Based Micelles for Cancer Therapy

**DOI:** 10.3389/fphar.2022.829590

**Published:** 2022-03-10

**Authors:** Ru Zhang, Yunying Jiang, Linkun Hao, Yang Yang, Ying Gao, Ningning Zhang, Xuecheng Zhang, Yimin Song

**Affiliations:** ^1^ Pharmaceutical Engineering Laboratory, Department of Pharmaceutical Engineering, Qingdao University of Science and Technology, Qingdao, China; ^2^ Pharmaceutical Engineering Laboratory, Colloge of Marines Life Science, Ocean University of China, Qingdao, China

**Keywords:** dual target, tumor actively targeted, micelles, reduction sensitive, doxorubicin hydrochloride (DOX), antitumor

## Abstract

In this study, a novel poly (lactic-co-glycolic acid) (PLGA)-based micelle was synthesized, which could improve the therapeutic effect of the antitumor drug doxorubicin hydrochloride (DOX) and reduce its toxic and side effects. The efficient delivery of DOX was achieved by active targeting mediated by double receptors and stimulating the reduction potential in tumor cells. FA-HA-SS-PLGA polymer was synthesized by amidation reaction, and then DOX-loaded micelles were prepared by dialysis method. The corresponding surface method was used to optimize the experimental design. DOX/FA-HA-SS-PLGA micelles with high drug loading rate and encapsulation efficiency were prepared. The results of hydrophilic experiment, critical micelle concentration determination, and hemolysis test all showed that DOX/FA-HA-SS-PLGA micelles had good physicochemical properties and biocompatibility. In addition, both *in vitro* reduction stimulus response experiment and *in vitro* release experiment showed that DOX/FA-HA-SS-PLGA micelles had reduction sensitivity. Molecular docking experiments showed that it can bind to the target protein. More importantly, *in vitro* cytology studies, human breast cancer cells (MCF-7), human non-small cell lung cancer cells (A549), and mouse colon cancer cells (CT26) were used to demonstrate that the dual receptor-mediated endocytosis pathway resulted in stronger cytotoxicity to tumor cells and more significant apoptosis. In and *in vivo* antitumor experiment, tumor-bearing nude mice were used to further confirm that the micelles with double targeting ligands had better antitumor effect and lower toxicity. These experimental results showed that DOX/FA-HA-SS-PLGA micelles have the potential to be used as chemotherapeutic drugs for precise tumor treatment.

## Introduction

Cancer is a common and frequently occurring disease threatening human health. At present, the traditional treatment of cancer mainly includes surgical resection, chemotherapy, radiotherapy, and immunotherapy. In recent years, photothermal, photoacoustic, and photodynamic therapies have also emerged. Among these treatments, chemical therapy is the most common strategy in cancer treatment (Chen et al., 2016; Zhang et al., 2017). DOX is a commonly used anticancer drug for anthraquinone antibiotics, which is widely used in the treatment of various solid tumors, lymphoma, acute and chronic leukemia, and other malignant tumors. However, it lacks specific selectivity to tumor cells, has low selectivity and short half-life when used alone, and also produces adverse reactions, such as cardiac toxicity, liver toxicity, and bone marrow suppression. Moreover, DOX is a liposoluble drug, which is easy to be lost after injection administration. It is metabolized rapidly in the blood and quickly eliminated by the kidneys, and the therapeutic effect is limited. These defects limit the clinical application of DOX (Cremers et al., 2017; Guo et al., 2020). Therefore, it is proposed that a drug delivery system that can transport DOX to the tumor site should be developed to achieve better antitumor effect and reduce adverse reactions. Due to the advantages that traditional delivery systems do not have, nano-drug delivery systems have been widely studied as drug carriers, and many nano-drug delivery systems have been developed, such as magnetic nanoparticles (Hosseinpour Moghadam et al., 2019), microemulsion (Lv et al., 2018; Wang et al., 2019), liposomes (Antimisiaris et al., 2021), gold nanoparticles (Lochbaum et al., 2021), platinum nanoparticles (Kawawaki et al., 2021), polymer micelles (Yang et al., 2021), etc. In these drug delivery systems, polymeric micelles are considered to be effective nanocarriers because they can encapsulate hydrophobic agents into their “core-shell” structures and deliver them to tumor regions through enhanced permeability and retention (EPR) effect. However, EPR effect only promotes the accumulation of micelles in tumors, and insufficient intracellular uptake is still a great challenge for effective chemotherapy (Bertrand et al., 2014; Fang et al., 2020; Maeda, 2021). The researchers used cancer cell-specific ligands as targets to make the delivery system have active targeting to improve the limitations of EPR effect, for example, folic acid (Scaranti et al., 2020), hyaluronic acid (Xiong et al., 2020), peptide (Cooper et al., 2021), transferrin (Yu et al., 2020), etc. They can easily enter cancer cells through receptor-mediated endocytosis, rather than relying solely on the EPR effect. Therefore, this method significantly enhances the antitumor effect and reduces the toxicity of the delivery system.

**FIGURE 1:**
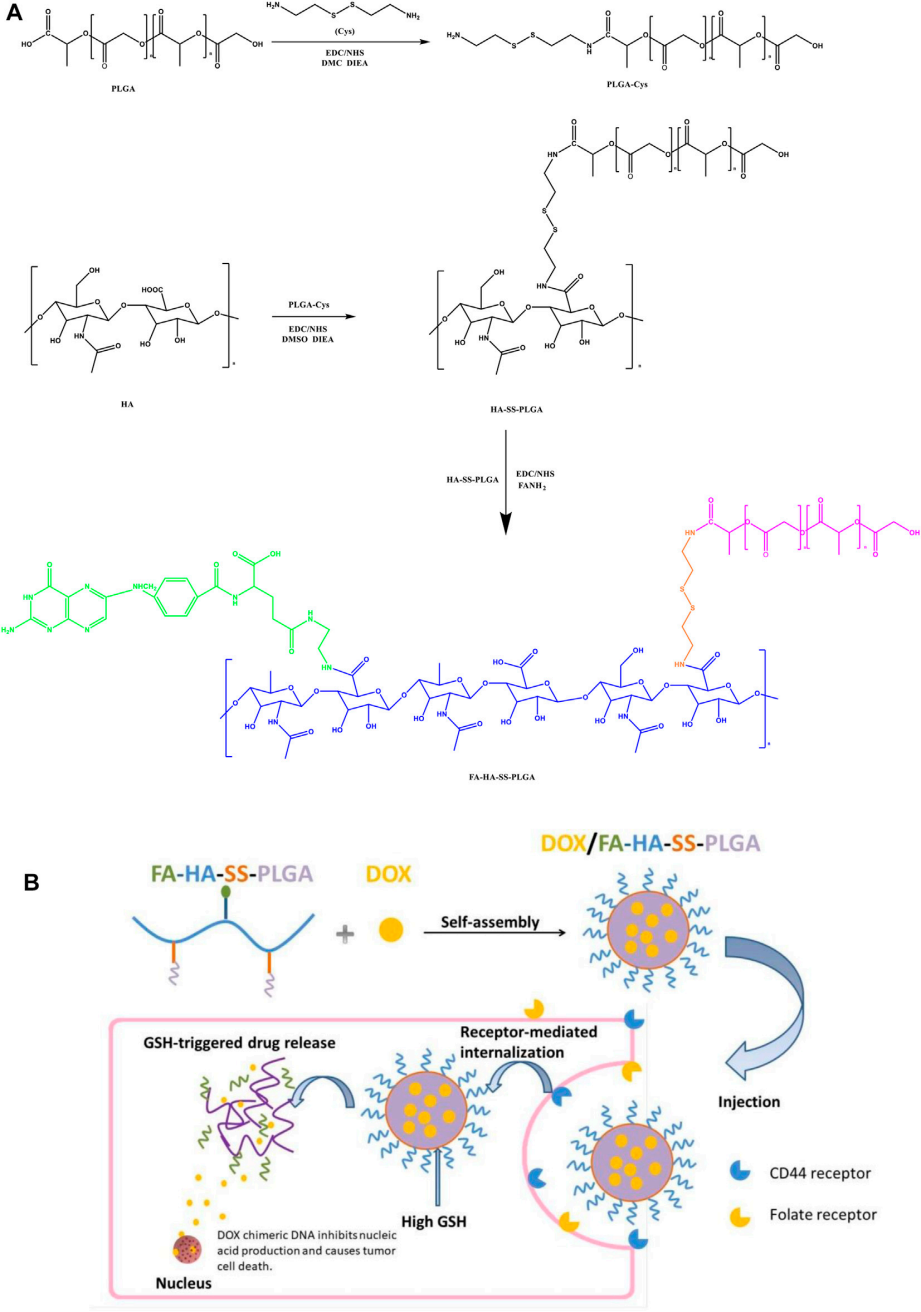
**(A)** Preparation process of folic acid (FA)-hyaluronic acid (HA)-SS-poly (lactic-co-glycolic acid) (PLGA). **(B)** Illustration self-assembly, tumor aggregation, and intracellular release of doxorubicin hydrochloride (DOX)/FA-HA-SS-PLGA micelles.

Hyaluronic acid (HA) is a natural acidic polysaccharide macromolecule, which is widely used in drug delivery due to its biodegradable and nontoxic properties, excellent biocompatibility, and easy chemical modification (Huang and Huang, 2018). The overexpression of HA-binding receptor CD44 on the surface of various cancer cells further expands the application of HA-based nanomedicine in active drug delivery targeting tumors (Jiang et al., 2012). In addition, the specificity of HA binding sites in organs is weak, so it shows a low accumulation in the liver, which promotes the accumulation of HA-based nano-drugs in tumor tissues (Poon et al., 2011; Lisha et al., 2014). However, aggregation and saturation of CD44 receptors may be one of the main factors leading to reduced targeting efficiency (Hu et al., 2016). Since the targeting ability of the nano-drug delivery system brought by a single targeted ligand is often limited, the introduction of different types of targeted ligands can make the nano-drug delivery system enter cells through different receptor-ligand-mediated endocytosis, which can improve the intracellularization ability of drugs to a certain extent (Zuo et al., 2017; Yin et al., 2012). Therefore, a nano-drug delivery system with a multitargeting function is designed to transport drug therapy, which provides a new research direction for cancer treatment (Zhang et al., 2021). It is reported that folic acid (FA) is an essential vitamin for base synthesis during cell proliferation, so FA is overexpressed on the surface of many tumor cells. However, the content of FA in human serum is very low, and cancer cells are likely to increase their uptake of FA by upregulating the expression of folate receptor. Cancer cells overexpressing folate receptors (FRs) include ovarian cancer, lung cancer, kidney cancer, endometrial cancer, breast cancer, brain cancer, colon cancer, and hematopoietic bone marrow cell carcinoma. According to the large upregulation of FR expression in tumor tissues, it is speculated that FA-modified therapeutic drugs can reduce the *in vivo* side effects and enhance the toxicity to tumor cells (Cagle et al., 2013). Therefore, FRs are considered as a tumor therapeutic target, which can provide an effective choice for targeted tumor therapy. More importantly, the simultaneous overexpression of CD44 receptor and FR in several tumor cells has been reported. For example, Manu M Joseph (Joseph et al., 2020), (Yan et al., 2019) used HA and FA to modify the drug delivery system, and proved that this dual-target drug delivery system could enhance cell uptake. However, in previous studies, there was still a problem that the drug encapsulated in the carrier cannot be released after tumor cells uptake such dual-target drug delivery system. The difficulty of drug release from the carrier was solved by means of environmental stimulus response in this study. It is reported that the stimulus response release system can control the release of drugs *in vivo* according to the external or internal environmental stimuli. The external environmental stimuli include temperature, magnetic field, light, or electric pulse, and the internal environmental stimuli include pH difference, enzyme concentration difference, or redox environment difference (Zhang et al., 2021), (Yatvin et al., 1978). As shown in Figure 1, according to the unique physiological conditions of tumor microenvironment, we used disulfide bonds to connect HA hydrophilic shell and PLGA hydrophobic core, and developed a reduction-responsive PLGA-based micelles. The glutathione (GSH) level of tumor cells is 7–10 times higher than that of normal tissues. This high reductive environment leads to the rapid fracture of micelles that reached the tumor site through thiol-disulfide bond exchange and drug encapsulated in the hydrophobic core of micelles released in tumor sites (Zhang et al., 2020). Therefore, disulfide bond is used as a connecting arm to achieve accurate and rapid drug release of micelles.

In this study, two functional ligands HA and FA with specific recognition of surface receptors were introduced simultaneously on PLGA for the first time, so that the nano-drug delivery system enters tumor cells through receptor-ligand-mediated endocytosis, which will greatly improve the drug intake ability of tumor cells (Ismail et al., 2015). In addition, disulfide bond was used as the connecting arm to enable micelles to release drugs accurately and rapidly under the stimulation of tumor reduction environment, and further enhance the targeting ability of drug delivery system. More importantly, PLGA has good biocompatibility and has been approved by the Food and Drug Administration (FDA). The monomers of its hydrolysis metabolites are lactic acid and glycolic acid, which are endogenous and can be simply metabolized into carbon dioxide and water by the tricarboxylic acid cycle. Its toxicity in drug delivery or application of biological materials can be ignored, but its poor hydrophilicity limits its application (Xu et al., 2015; Dong et al., 2018). Therefore, we selected PLGA as the hydrophobic core and loaded the antitumor drug DOX to synthesize amphiphilic DOX/FA-HA-SS-PLGA micelles. Then its redox sensitivity and drug release *in vitro* were studied under a high concentration of GSH, and its antitumor activity *in vitro* was studied by the MTT method. Finally, its antitumor effect *in vivo* and reducing side effects were verified in nude mice-bearing tumor.

## Materials and Methods

### Materials

PLGA (75:25) was purchased from Baide Chemical Reagent Co., Ltd. (Jiangsu, China). N-hydroxysuccinimide (NHS), 1-ethyl-3 (3-dimethylaminopropyl) carbodiimide (EDC), cystamine (CYS), ethylenediamine (EDA), dicyclohexylcarbodiimide (DCC), and GSH were purchased from Chemical Reagent Co., Ltd. (Shanghai, China). HA (5000Da) was purchased from the Furuida Pharmaceutical Group (Shandong, China). FA was purchased from Bomei Biotechnology Co., Ltd. (Tianjin, China). MCF-7 cells, A549 cells, and CT26 cells were purchased from Shanghai Pharmaceutical Research Institute. All other chemicals and reagents were of analytical reagent grade.

### Preparation of Hyaluronic acid-SS-Poly (Lactic-co-Glycolic Acid) and Ethylenediamine-SS-Poly (Lactic-co-Glycolic Acid)

HA-SS-PLGA was synthesized by grafting PLGA onto the skeleton of HA through EDC/NHS coupling reaction. First, PLGA-CYS was synthesized by the acylation reaction between a CYS amine and the terminal carboxyl of PLGA. In 20 ml of dichloromethane, 0.5 mmol NHS, 0.5 mmol EDC, and 0.05 mmol PLGA were activated for 2 h. Then excessive CYS and N,N-diisopropylethylamine (DIEA) were added and stirred at ambient temperature overnight. The obtained product was precipitated in cold ether and washed with cold methanol to remove the unreacted reactants. The product PLGA-CYS was dried in vacuum to remove the residual solvent, which could be used without further treatment. HA-SS-PLGA was synthesized by acylation reaction between amino group of PLGA-CYS and terminal carboxyl group of HA. In 20 ml of dimethyl sulfoxide (DMSO), 0.6 mmol NHS, EDC, and 0.04 mmol HA were activated for 2 h, and then the prepared PLGA-CYS was added for overnight reaction. The obtained precipitate was dialyzed in deionized water for 48 h and freeze dried to obtain HA-SS-PLGA. As a control, nonreducing HA-EDA-PLGA was synthesized using EDA instead of CYS as described above. The chemical structure of the obtained product was detected by Fourier transform infrared spectrometer (TENSOR, Bruker, Germany).

### Preparation of Folic acid-Hyaluronic acid-SS-Poly (Lactic-co-Glycolic Acid)

FA was aminated and modified according to published literature (Dong et al., 2018). DCC, NHS, and FA were dispersed in DMSO in the ratio of 1.5:1.5:1 (mol), reacted at 50°C for 6 h, then excessive EDA and pyridine were added, and reacted at ambient temperature for12 h. The reaction solution was precipitated three times in ether, and the light yellow powder (FANH_2_) was obtained by freeze drying. The prepared HA-SS-PLGA, EDA, and NHS were dispersed in formamide at a ratio of 1.5:1.5:1 (mol), and the hydroxyl was activated in ice bath for 2 h. Then FANH_2_ was added and reacted at room temperature for 24 h. The reaction solution was dialyzed for 3 days and finally freeze dried to obtain the product FA-HA-SS-PLGA polymer. FA-HA-EDA-PLGA was prepared by the above method.

### Study on Oxidation–Reduction

FA-HA-SS-PLGA (30 mg) and FA-HA-EDA-PLGA (30 mg) were dispersed in phosphate buffer solution (PBS, pH 7.4) and ultrasonically treated at 100 W for 15 min in ice bath. The solution was incubated with GSH at various concentrations in a constant temperature vibration incubator at 37°C for 24 h. Within 5 h, the size changes were studied by multifunctional particle size analyzer (CAMSIZER XT, Guangzhou Aiwei Instrument Technology Co., Ltd.) every 1 h.

### Preparation and Characterization of Micelles Loaded With Doxorubicin Hydrochloride

#### Process Optimization

Considering the thermodynamic instability of PLGA (He et al., 2014), this experiment adopted the dialysis method without heating to prepare DOX/FA-HA-SS-PLGA micelles by encapsulating hydrophobic DOX into the hydrophobic core of the polymer. DOX was accurately weighed and ultrasonically dispersed in 4 ml of deionized water. DOX/FA-HA-SS-PLGA micelles were prepared by adding a certain amount of FA-HA-SS-PLGA into DOX solution, stirring for 4 h, dialysis for 12 h, and ultrasonic dispersion for 15 min.

Response surface methodology (RSM) was used to optimize the nanoparticles. The drug loading ratio (A, DOX:FA-HA-SS-PLGA = 1:10, 2:10, 4:10), stirring speed (B), and stirring time (C) were set as independent variables, and drug loading (R_1_) and encapsulation efficiency (R_2_) were set as response values. The preparation process was optimized by the Box–Behnken method using the Design-Expert software.

##### Determination of Drug Loading Rate and Entrapment Efficiency

DOX/FA-HA-SS-PLGA (10 mg) was dissolved in an appropriate amount of saturated (NH_4_)_2_SO_4_ solution and subjected to ultrasonic oscillation for 10 min. The solution was centrifuged (10,000 r/min, 15 min), dried at room temperature, and dissolved in deionized water. The dialysis was performed under magnetic stirring for 72 h (retention value 1,000 D), and the dialysis solution was replaced every 12 h. The prepared DOX/FA-HA-SS-PLGA (10 mg) was dissolved in deionized water (10 ml), and its absorbance was measured by UV spectrophotometer (752N, Shanghai Jingke Instrument Co., Ltd.) at 480 nm. The concentration of DOX solution was calculated according to the concentration-absorption standard curve. All experiments were repeated three times, and the results were expressed as mean ± standard deviation.

The calculation formulas of loading capacity (LC) and encapsulation efficiency (EE) are as follows.

$$\mathrm{LC}\left(\%\right)=\frac{M_{f}}{M_{s}}\times 100\%$$

$$\mathrm{EE}\left(\%\right)=\frac{M_{f}}{M_{n}}\times 100\%$$

$M_{f}$ was the mass of DOX encapsulated into the carrier, and $M_{n}$ was the mass of DOX required to prepare the corresponding DOX/FA-HA-SS-PLGA drug delivery system. The quality of DOX/FA-HA-SS-PLGA drug delivery system was weighed by $M_{s}$ .

#### Characterization of Particle Size, Surface Potential, and Morphology

Multifunctional particle size analyzer (Guangzhou Aiwei Instrument Technology Co., Ltd.) was used to measure the particle size and Zeta potential of PLGA, PLGA-SS-HA, FA-HA-SS-PLGA, and DOX/FA-HA-SS-PLGA (15 mg each). Scanning electron microscopy (SEM, Hitachi, Japan) and transmission electron microscopy (TEM, Hitachi, Japan) were used to observe their surface morphology.

### Hydrophilicity Experiment

PLGA, HA-SS-PLGA, FA-HA-SS-PLGA, and DOX/FA-HA-SS-PLGA (10 mg each) were dissolved in 1 ml of dichloromethane, and then the solution was evenly spread on the glass slide. A thin film with a thickness of about 10 μm was formed on the glass surface after solvent evaporation, and then dried at room temperature for 24 h. The titration method was used to measure the water contact angle of the polymer, five times per sample.

PLGA, HA-SS-PLGA, FA-HA-SS-PLGA, and DOX/FA-HA-SS-PLGA (10 mg each) were ultrasonically dispersed in 5 ml of deionized water to observe their solubility and stability in water.

#### Critical Micelle Concentration

The CMC values of amphiphilic polymers and drug-loaded micelles were determined by pyrene fluorescence spectroscopy (Ashjari et al., 2012). The acetone solution with a concentration of 4 × 10^−7^ mol/L pyrene was precisely prepared. Subsequently, a series of micelle samples with different concentrations were prepared. The acetone solution was taken, respectively, and added into a 10-ml volumetric flask. The stable micelle pyrene solutions with concentrations of 200, 80, 40, 20, 10, 5, 2.5, 1, 0.5, and 0.25 μg/ml were accurately prepared. The first peak (I_1_ = 374 nm) and the second peak (I_3_ = 395 nm) were recorded to calculate the ratio (I_1_/I_3_), and the excitation ratio of I_1_/I_3_ was used to determine the CMC value (Wei et al., 2019).

### *In vitro* Release

The dried DOX/FA-HA-SS-PLGA was weighed as 5 mg in a conical flask, and 30 ml of PBS (pH 7.4) was used as the release medium to add GSH (0 mM or 10 mM) with different concentrations to the buffer solution. The speed was adjusted to 60 r/min, and the bath temperature was 37 ± 0.5°C. At a specific time interval (0.5, 1, 2, 3, 4, 5, 6, 8, 12, 18, 24, 36, 48, 60, 72 h), 1 ml of sample was removed by a liquid gun and placed in a centrifuge tube. The supernatant was centrifuged at 4°C for 30 min at 14,000 r/min. The absorbance of the supernatant was determined by UV spectrophotometer. The content of DOX in the supernatant was determined by comparing the concentration-absorption standard curve of DOX, and the cumulative release rate of the drug was calculated.

### Molecular Docking of Drug-Loaded Micelles With Target Proteins

Two target proteins, CD44 receptor and folate receptor, were downloaded from the PDB database. When selected, human protein is preferred, and the original ligand has high similarity with the active component to be docked. Then the Pymol 2.3.0 software was used to remove protein crystal water, original ligand, and so on. The protein structure was imported into Auto Docktools (v1.5.6) for hydrogenation, calculation of charge, distribution of charge, and specified atomic type. Finally, Auto Dock Vina1.1.2 software was used for docking.

### Hemolysis Test

Blood was taken from rat orbit and placed in heparinized EP tubes with the same volume of saline. The mixture was placed in a centrifuge, (3,000 r/min, 5 min), the supernatant was discarded, the same volume of saline was continuously added, centrifugation, to the supernatant clear, precipitation is red blood cells. A 2% red blood cell suspension with normal saline was prepared. The same volume of 2% red blood cell suspension was added into the blank micelle solution and drug-loaded micelle solution with a concentration of 0.125 –2 mg/ml. The mixture was evenly mixed and placed in a 37°C water bath. After 2 h, the supernatant was centrifuged at 3,000 r/min for 10 min and 200 μl after centrifugation. The absorbance (OD) was measured by UV spectrophotometer at 545 nm. In addition, red blood cells were treated with purified water and 0.9% saline as positive and negative controls. All data were the average of three repeated tests. Hemolytic rate (HR, %) could be calculated as follows.

$$\mathrm{HR}=\frac{OD_{t}-OD_{\mathrm{nc}}}{OD_{\mathrm{PC}}-OD_{\mathrm{nc}}}\times 100\%$$

In the above formula, ***OD***_***t***_ was the absorbance of the experimental sample, ***OD***_***nc***_ was the absorbance of negative control sample, and ***OD***_***pc***_ was the absorbance of positive control sample.

### Study on Antitumor Activity *in vitro*

MCF-7, A549, and CT26 in logarithmic growth phase were digested and dispersed by trypsin and then diluted with DMEM medium containing 10% fetal bovine serum into a single-cell suspension with a cell density of 5 × 10^4^ cells/ml, which was inoculated into a 96-well cell culture plate. The cells were cultured in a 5% CO_2_ incubator at 37°C for 24 h. Different types of DOX-loaded micelle solutions (DOX-equivalent dose: 0.5, 1, 2.5, 5, and 10 μg/ml) were added after cell adherent growth and cultured for 24 and 48 h.

The untreated blank micelles were used as the negative control, and DOX was used as the positive control. Three parallel groups were set for each well. DMSO (0.1 ml) was added to each well to dissolve the purple precipitate formazan, which was fully dissolved by constant temperature oscillation for 30 min. The absorbance at 570 nm was measured by a microplate reader (ELX800, Shanghai Jingke Instrument Co., Ltd), and the cell survival rate was calculated according to the following formula:

$$\mathrm{Cell activity}=\frac{OD_{\mathrm{test}}}{OD_{\mathrm{control}}}\times 100\%$$

***OD***_***test***_ was the absorbance value measured after culturing cells in the experiment, and ***OD***_***control***_ was the absorbance of the cultured cells.

The apoptosis rate of MCF-7 cells induced by various drug-loaded formulations was quantitatively evaluated by Annexin V-FITC/PI staining method. The cells were cultured with DOX-PLGA, DOX/FHA-SS-PLGA, DOX/FA-SS-PLGA, and DOX/FA-HA-SS-PLGA micelles at an equivalent DOX concentration (120 μg/ml). After 12 h, the cells were harvested for Guava Nexin Reagent staining. Finally, the stained cells were analyzed by flow cytometer (BD FACSCALIBUR, USA).

### Cellular Uptake

With respect to the evaluation of cellular uptake of micelles, fluorescein isothiocyanate (FITC), as a substitute for DOX, was entrapped in polymeric micelles in line with the protocol of PTX-loaded micelles. When the MCF-7 cells reached approximately 80% confluence, various FITC-loaded formulations at equivalent FITC concentration of 0.5 μg/ml were added to incubate with cells for additional 2 h. The cell morphology, fluorescence intensity, and flow cytometry were observed and compared under inverted fluorescence microscope.

### Antitumor Effect *in vivo*

#### Animal and Tumor Xenotransplantation Model

Nude mice (aged 5–6 weeks, weighing 18–22 g) were obtained from Yushan Animal Farm, Qingdao, China. All animal care and experiments were conducted according to the “Laboratory Animal Care and Use Guide,” designated by the National Institutes of Health. The Ethics Committee of Qingdao University of Science and Technology approved the experiment. MCF-7 cells (1 × 10^7^ cells/mouse) were inoculated subcutaneously in nude mice. Tumor volume was monitored by caliper measurement

#### Antitumor effect *in vivo*

The tumor-bearing nude mice were randomly divided into five groups when the tumor volume increased to about 100 mm^3^, with five mice in each group (*n* = 5). Normal saline, PLGA-DOX, DOX/HA-SS-PLGA micelles, DOX/FA-SS-PLGA micelles, and DOX/FA-HA-SS-PLGA micelles were injected by tail vein at a standard dose of 8.0 mg/kg (DOX-equivalent dose), one injection every 2 days, five times. The body weight and tumor volume of mice were recorded. Then the mice were sacrificed, and the tumors were removed and weighed. Tumor weight inhibition rate (IR) was calculated by the following equation:

$$\mathrm{IR}=\frac{W_{\mathrm{control}}-W_{\mathrm{test}}}{W_{\mathrm{test}}}$$

***W***_***control***_ and ***W***_***test***_ represent the average tumor weight of the normal saline group and treatment group, respectively.

#### Pharmacokinetics Study

DOX, PLGA-DOX, DOX/HA-SS-PLGA micelles, DOX/FA-SS-PLGA micelles and DOX/FA-HA-SS-PLGA micelles were injected into mice by tail vein at a dose of 8.0 mg/kg (DOX-equivalent dose), respectively. At predetermined time points (0.1, 0.2, 0.5, 1, 2, 5, 10, 15, 20, and 25 h), blood samples were collected into previously weighted tubes. Subsequently, 0.15 ml (1% v/v Triton X-100) of lysate was added to the plasma, followed by ultrasonication for 3 min, and the DOX content was determined by HPLC. The two-phase half-life was obtained by quadratic exponential decay fitting, and the calculation formula was as follows:

$$y=A_{1}\times \exp\frac{-x}{t_{1}}+A_{2}\times \exp\frac{-x}{t_{2}}$$

$$t_{1/2,\alpha }=0.693\times t_{1}$$

$$t_{1/2,\beta }=0.693\times t_{2}$$

***t***_***1/2,α***_ was the half-life of the dispersed phase, and ***t***_***1/2,β***_was the half-life of the eliminated phase.

### Investigation on the Stability of Drug-Loaded Micelles of Doxorubicin Hydrochloride/ Folic Acid-Hyaluronic Acid-SS-Poly (Lactic-co-Glycolic acid)

#### Influencing Factors Experiment

In a random batch of 20% DOX/FA-HA-SS-PLGA drug delivery system, 15 mg of dry DOX/FA-HA-SS-PLGA was taken for high temperature experiment (40°C ± 2°C), high humidity experiment (90% ± 5%), and strong light experiment (4,500 ± 5,00 lx), respectively. The release and UK content of DOX/FA-HA-SS-PLGA drug delivery system were detected on the 0, 5th, and 10^th^ days.

#### Accelerated Stability Experiment

In the 20% DOX/FA-HA-SS-PLGA drug delivery system prepared in three batches (numbered as 1, 2, and 3), 15 mg of dry DOX/FA-HA-SS-PLGA was taken from each batch and placed for 6 months at 40°C ± 2°C and 75% ± 5% relative humidity. Samples were taken once in the 0th, 1st, 2nd, 3rd, and 6th months during the test to detect the release rate and UK content of the DOX/FA-HA-SS-PLGA drug delivery system.

#### Long-Term Stability Experiment

In the 20% DOX/FA-HA-SS-PLGA drug delivery system prepared in three batches (numbered as 1, 2, 3), 15 mg of dry DOX/FA-HA-SS-PLGA was taken from each batch and placed for 18 months at 25°C ± 2°C and 60% ± 10% relative humidity. Samples were taken once in the 0th, 3^rd^, 6^th^, 9^th^, 12^th^, and 18^th^ months of the test period to detect the release rate and UK content of the DOX/FA-HA-SS-PLGA drug delivery system.

### Statistical Analysis

All quantitative data are shown as the mean ± SEM. Statistical significance was determined with one-way analysis of variance (ANOVA) and Student’s t-test at 95% confidence levels.

## Results and Discussions

### Synthesis and Characterization of Poly (Lactic-co-Glycolic Acid)-Cystamine, Hyaluronic acid-SS-Poly (Lactic-co-Glycolic Acid) and folic acid-Hyaluronic acid-SS-Poly (Lactic-co-Glycolic acid)

**FIGURE 2:**
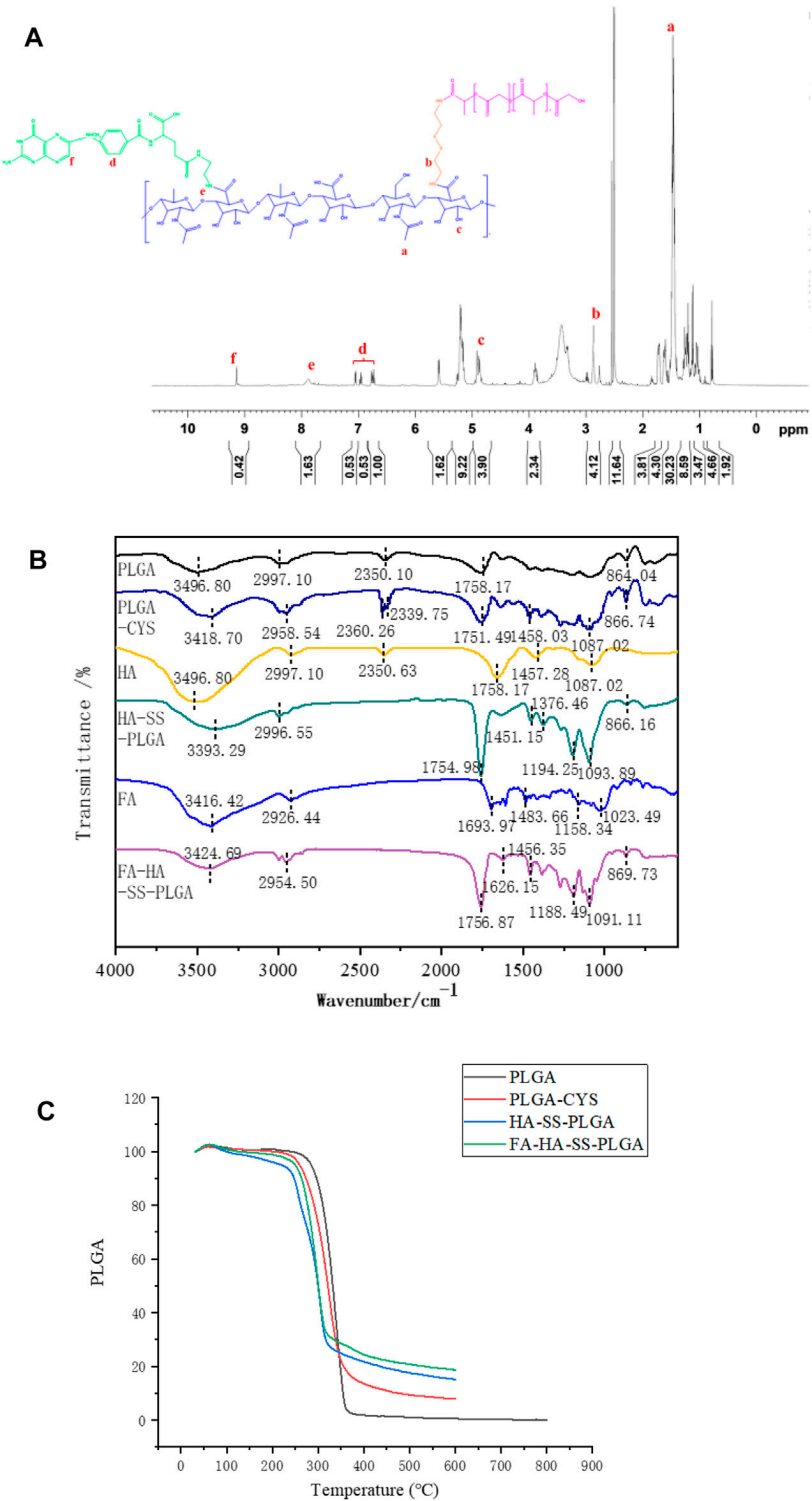
**(A)** ^1^H NMR spectrum of FA-HA-SS-PLGA. **(B)** Infrared spectra of PLGA, PLGA-CYS, HA, HA-SS-PLGA, FA, and FA-HA-SS-PLGA. **(C)** Thermogravimetric analysis curves of PLGA, PLGA-CYS, HA-SS-PLGA, and FA-HA-SS-PLGA.

The ^1^H NMR spectra of FA-HA-SS-PLGA are shown in Figure 2A. Peak a (1.4 ppm) and peak c (4.9 ppm) were the hydrogen signals in the methyl and hydroxyl groups in the HA structure, respectively. Peak b (2.8 ppm) was the methylene hydrogen signal in cystamine structure. Peaks d (6.7, 6.9, 7.1 ppm) and f (9.2 ppm) were the characteristic proton peaks of FA. Peak e was the hydrogen signal on the amide bond of FA and HA. As shown in Figure 2B, in the infrared data of PLGA-CYS, the nonplanar swing vibration absorption peak of –CH_2_– in the –SCH_2_– structure was at 1,087.02 cm^−1^, which was an important signal indicating the existence of sulfur atoms. In the diagram of HA-SS-PLGA, the peak at 1,376.46 cm^−1^ was attributed to the associated C–N stretching vibration, which indicated that the amide bond was introduced into HA to form HA-SS-PLGA polymer. Similarly, the infrared spectra of FA-HA-SS-PLGA showed that there was a set of miscellaneous peaks at about 3,424.69 cm^−1^, which was caused by the amide bond formed by the amidation reaction between FA and HA in HA-SS-PLGA. The above description proved the successful synthesis of FA-HA-SS-PLGA polymer.

In Figure 2C, the thermogravimetric curves of PLGA-CYS, HA-SS-PLGA and FA-HA-SS-PLGA were similar to those of PLGA. All the four samples entered the accelerated decomposition stage at about 230°C, which was the main decomposition stage of lattice material. The thermal decomposition rates of HA-SS-PLGA and FA-HA-SS-PLGA decreased at 320°C because both structures contained disulfide bonds (–S–S–), which was a relatively stable covalent bond. This made the thermal stability of HA-SS-PLGA and FA-HA-SS-PLGA slightly higher than that of PLGA and PLGA-CYS, which proved that there was indeed a disulfide bond structure in the prepared HA-SS-PLGA and FA-HA-SS-PLGA.

### Oxidative Reductivity of Polymers

**FIGURE 3:**
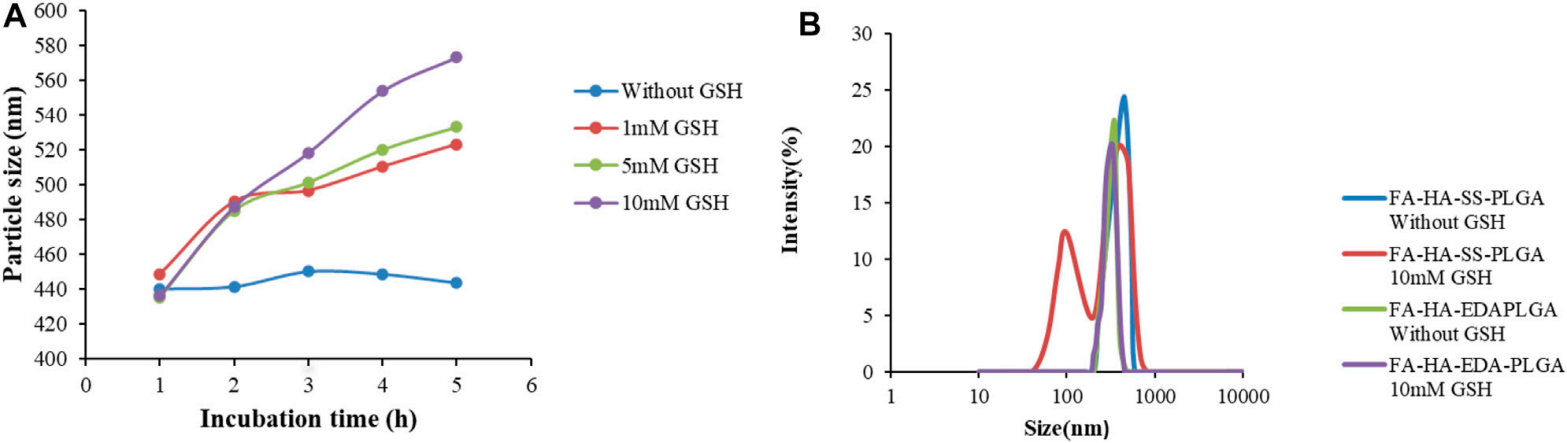
The change in particle size and morphology of reduced sensitive FA-HA-SS-PLGA polymer with glutathione (GSH) concentration. **(A)** The particle size of HA-SS-PLGA in the presence of GSH (0, 1, 5, and 10 mM). **(B)** The particle size distribution of FA-HA-SS-PLGA and FA-HA-EDA-PLGA cultured in 10 mM GSH or without GSH for 24 h.

The DLS instrument was used to monitor the effect of different concentrations of GSH (0, 1, 5, 10 mM) on particle size within 5 h to prove the reduction sensitivity of FA-HA-SS-PLGA polymer. As shown in Figure 3A, the particle size of FA-HA-SS-PLGA increased significantly with the increase in GSH concentration and time, especially at the highest GSH concentration (10 mM). In addition, the particle size of FA-HA-SS-PLGA polymer was doubled within 2 h. On the contrary, in the absence of GSH, the particle size of FA-HA-SS-PLGA polymer remained almost unchanged for all incubation periods within 5 h, so they could remain intact during prolonged blood circulation.

The particle size distribution of FA-HA-SS-PLGA and FA-HA-EDA-PLGA FA-HA-SS-PLGA and FA-HA-EDA-PLGA cultured in 10 mM GSH or without GSH for 24 h was measured to prove the reductive sensitive fracture of disulfide bond (–S–S–) in micelles. As shown in Figure 3B, the size distribution of FA-HA-EDA-PLGA remained unchanged after incubation with PBS (pH 7.4) for 24 h, regardless of whether GSH was present. On the contrary, the presence of 10 mM GSH resulted in a significant change in the size distribution of FA-HA-SS-PLGA. The trigger fracture of disulfide bonds in polymers depended on the amount of GSH around them, indicating that the disulfide bonds in amphiphilic drug-loaded micelles DOX/FA-HA-SS-PLGA can react to trigger fracture in the cytoplasm of tumor reductive environment. The fracture of the connecting arm between the hydrophilic group and the hydrophobic group led to the instability of the hydrophobic core, and the drugs encapsulated in the hydrophobic core can be released. Therefore, it was expected to produce a rapid inhibitory effect on tumor through drug-triggered release. Overall, these findings suggested that reduction-sensitive FA-HA-SS-PLGA polymers are suitable for delivery of anticancer drugs to tumor sites.

### Preparation and Characterization of Doxorubicin Hydrochloride/Folic acid-Hyaluronic Acid-SS-Poly (Lactic-co-Glycolic Acid) micelles

#### Experimental Analysis of Drug Loading Rate Optimization

The Design Export 8.0 software was used to perform variance analysis and significance test on the data in response to surface regression fitting (Supplementary Table S1). The quadratic regression fitting model is obtained, and the formula is as follows.

R_1_ = 21.44 + 0.48A + 0.063B-0.038C + 0.025AB + 0.025AC − 0.2BC−3.2A^2^−1.27 B^2^−1.37C^2^.The detailed results are shown in Supplementary Table S1.

**FIGURE 4:**
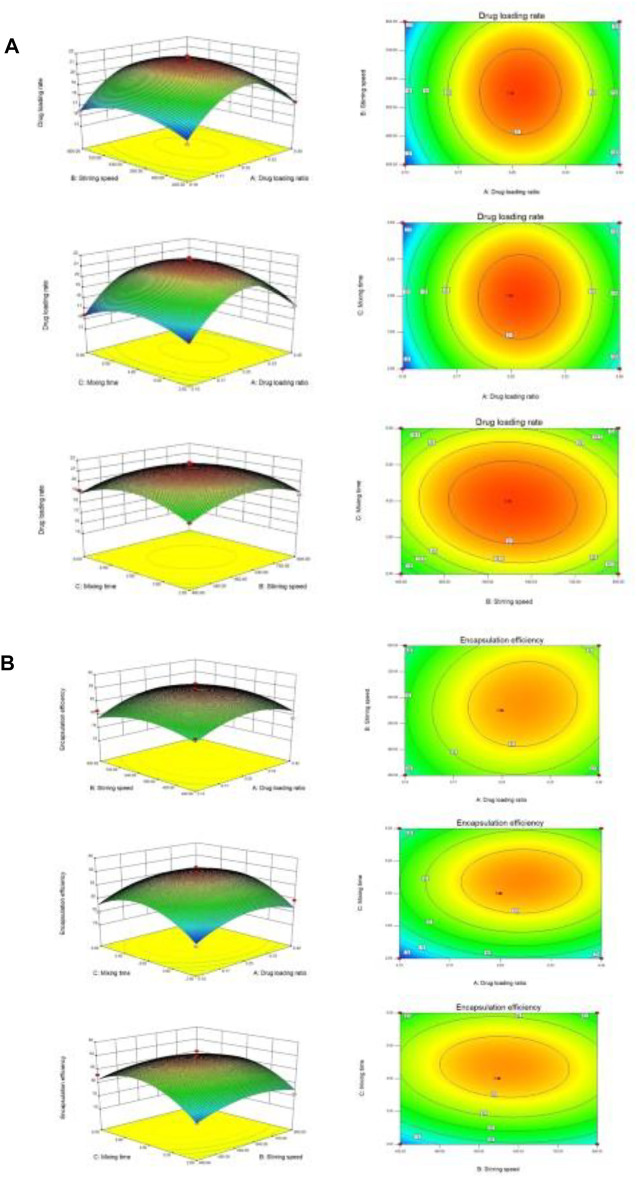
3-D response surface plots. **(A)** The influence of independent variable factors on drug loading. **(B)** The influence of independent variable factors on encapsulation efficiency.

The results of variance analysis showed that the *p*-value of the model was 0.05, which was not significant, indicating that the fitting degree of the model was good, and this model can be used to optimize the preparation process. It can be seen from the above table that the order of significant effects on drug loading rate was A (drug-loading ratio) > B (stirring speed) > C (stirring time). The response surface of three variables to drug loading rate was obtained by the Design Expert 8.0 software as shown in Figure 4A.

#### Experimental Analysis of Encapsulation Efficiency Optimization

The Design Export 8.0 software was used to perform variance analysis and significance test on the data in response to surface regression fitting Supplementary Table S2. The quadratic regression fitting model is obtained, and the formula is as follows. R_2_ = 83.32 + 0.75A + 0.21B + 0.99C + 0.27AB + 0.075AC−0.3BC−1.99A^2^−1.51B^2^−2.86C^2^. The detailed results are shown in Supplementary Table S2.

The results of variance analysis showed that the *p*-value of the model was 0.05, which was not significant, indicating that the fitting degree of the model was good, and this model can be used to optimize the preparation process. It can be seen from the above table that the order of significant effects on encapsulation efficiency was C (stirring time) > A (drug-loading ratio) > B (stirring speed). The response surface of three variables to encapsulation efficiency was obtained by the Design Expert 8.0 software as shown in Figure 4B.

#### Best Technology and Reproducibility Experiments

The Design Expert 8.0 software was used to analyze the data. The drug loading rate and encapsulation efficiency were used as evaluation indexes. The results showed that the best preparation process was obtained when the drug loading ratio was 0.32, the stirring speed was 612.49 r/min, and the stirring time was 4.21 h. In the actual operation, the drug loading ratio was adjusted to 0.3, the stirring speed was610 r/min, and the stirring time was 4.2 h. Under the above conditions, the drug loading rate was 21.0%, and the encapsulation efficiency was 83.6%. The drug loading rate was 21.1 (±0.1)%, and the encapsulation efficiency was 83.2 (±0.2)%, which was basically consistent with the predicted value of the model, so the model obtained by the software was reliable.

#### Properties and Morphology of Micelles

The prepared polymers and various properties of DOX micelles are summarized in Table 1. The diameters of PLGA-CYS and HA-SS-PLGA were larger than their corresponding blanks, respectively. This phenomenon may be due to the continuous increase in polymer diameter caused by the successive bonding of various groups to PLGA. The absolute values of the Zeta potential of PLGA-CYS, HA-SS-PLGA, FA-HA-SS-PLGA, and 20% DOX/FA-HA-SS-PLGA gradually decreased because the negative carboxyl (–COOH) in PLGA and HA was consumed in the reaction.

**TABLE 1:** Physical and chemical properties of micelles (*n* = 3, $\bar{x}$ ± SEM).

Type of samples	Zeta potential (mV)	Particle size (nm)	PDI
Poly (lactic-co-glycolic acid) (PLGA)-cystamine (CYS)	−37.2 ± 1.2	160.87 ± 1.32	0.175 ± 0.045
Hyaluronic acid (HA)-SS-PLGA	−35.2 ± 0.8	224.53 ± 1.41	0.154 ± 0.098
Folic acid (FA)-HA-SS-PLGA	−27.3 ± 0.7	440.67 ± 1.23	0.197 ± 0.047
Doxorubicin hydrochloride (DOX)/FA-HA-SS-PLGA	−26.3 ± 1.5	307.47 ± 1.50	0.174 ± 0.033

**FIGURE 5:**
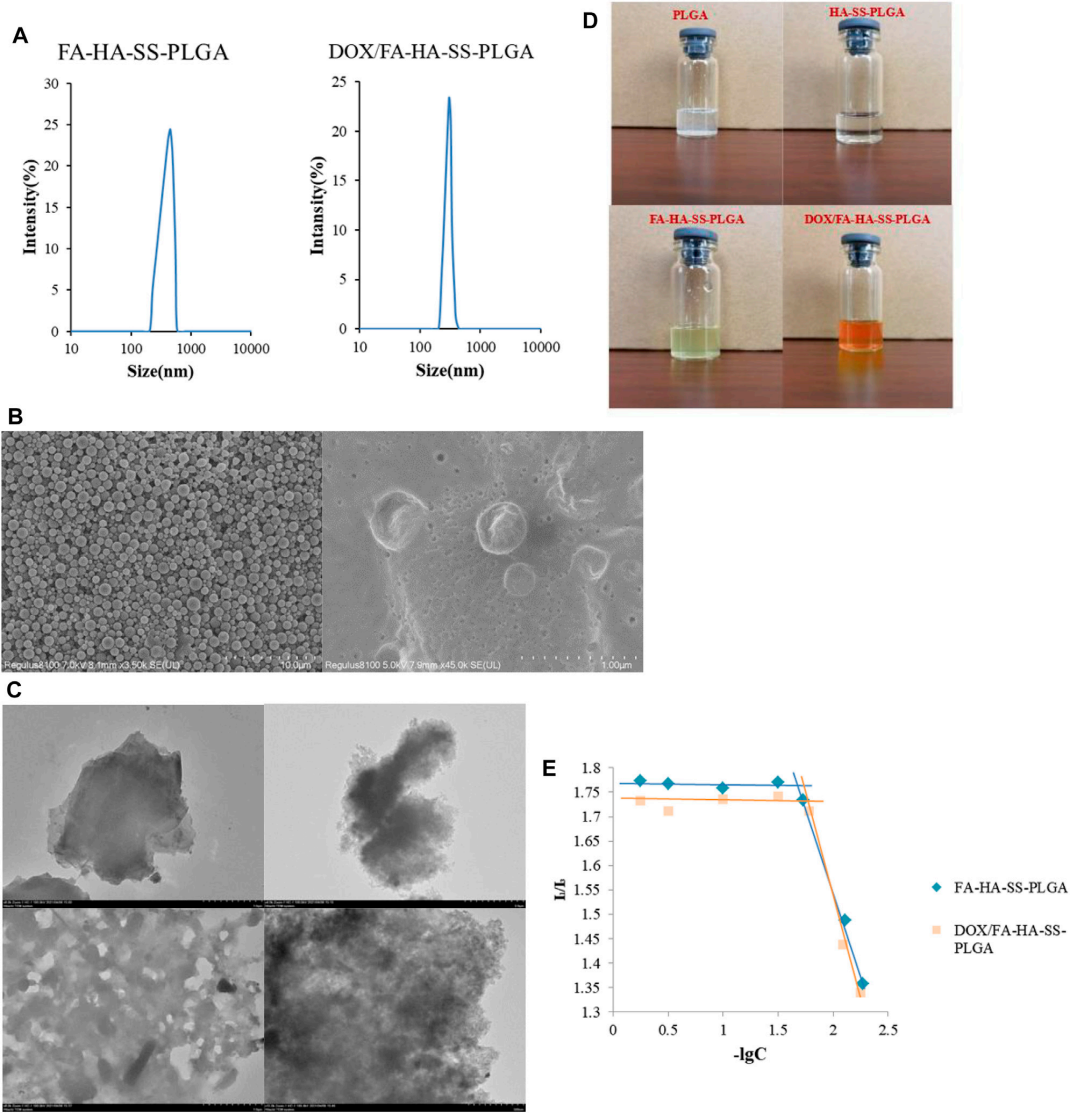
Properties of the prepared polymers and micelles.**(A)** Size distribution of FA-HA-SS-PLGA and DOX/FA-HA-SS-PLGA. **(B)** Scanning electron microscopy (SEM) micrographs of FA-HA-SS-PLGA and DOX/FA-HA-SS-PLGA. **(C)** Transmission electron microscopy (TEM) micrographs of PLGA, HA-SS-PLGA, FA-HA-SS-PLGA, and DOX/FA-HA-SS-PLGA. **(D)** Dispersion of various polymers in water after 72 h. **(E)** Critical micelle concentrations of FA-HA-SS-PLGA and DOX/FA-HA-SS-PLGA. The data were expressed as mean ± SEM (*n* = 3).

Figure 5A showed that the two materials were nearly spherical in structure and have narrow size distribution. As shown in Figure 5B, FA-HA-SS-PLGA was found to have a gap between molecules, and the surface was relatively smooth and without impurities. However, there was a layer of blurred substance attached to the surface of DOX/FA-HA-SS-PLGA and became uneven, indicating that DOX was successfully loaded into FA-HA-SS-PLGA.

The morphological structures of PLGA, HA-SS-PLGA, FA-HA-SS-PLGA and DOX/FA-HA-SS-PLGA were detected by TEM. As shown in Figure 5C, the PLGA without any treatment had a clear edge, smooth surface, and no obvious particles. However, the boundary of the HA-SS-PLGA polymer prepared after its connection with HA became blurred, the lamellar thickness increased, and the transmittance decreased. It was speculated that the disulfide bond (–S–S–) acted as a connecting arm to connect HA with PLGA. After FA was reconnected, the transmittance of the polymer increased. The polymer formed a hollow hydrophobic core structure, which was a necessary condition for the successful loading of DOX. After loading the drug by dialysis, it was observed that the void structure basically disappeared, and the material surface tended to be smooth, which was because DOX was loaded into the void of the polymer FA-HA-SS-PLGA. This proved the successful preparation of DOX/FA-HA-SS-PLGA micelles.

The hydrophilicity of amphiphilic polyester copolymer was evaluated by measuring the water contact angle. The contact angles of HA-SS-PLGA and FA-HA-SS-PLGA were 67° and 43°, which were both hydrophilic polymers. Compared with them, the contact angle of PLGA was 98°, which was a hydrophobic substance (Taubner et al., 2020). Therefore, PLGA copolymers with more lactic acid components have low hydrophilicity (Wang et al., 2005), (Ryu et al., 2021). The ratio of PLGA lactic acid to glycolic acid used in this experiment was 75:25, which was confirmed to be hydrophobic by measurement. Although the hydrophobic property of PLGA limited its application, experiments showed that PLGA can improve its hydrophilicity by connecting with other water-soluble substances. The dispersion of substances in water is shown in Figure 5D. PLGA was difficult to disperse uniformly in deionized water and had obvious precipitation and flocculent suspension. After connecting HA and FA, it can be stably dispersed in water, which was speculated to be related to the large amount of hydroxyl in the molecular structure of HA and FA connected by PLGA.

When the concentration of the polymer is greater than CMC, pyrene preferentially enters the hydrophobic microstructure of the polymer, the change of molecular photophysical properties (Waters et al., 2020). Therefore, with the increase in polymer concentration, the fluorescence intensity of pyrene emission spectrum has a mutation, which indicates the formation of micelles. As shown in Figure 5E, the critical micelle concentrations of FA-HA-SS-PLGA and DOX/FA-HA-SS-PLGA were 25.68 ± 2.31 and 23.31 ± 1.91 μg/ml, respectively, by plotting the corresponding concentration pairs of I_1_/I_3_ and taking values at the turning point of the graph line. The results showed that the CMC values of FA-HA-SS-PLGA and DOX/FA-HA-SS-PLGA were low, indicating that the drug-loading system could maintain its stability in the process of reaching the target through blood circulation (Kang et al., 2019).

### *In vitro* Drug Release of Drug-Loaded Micelles

**FIGURE 6:**
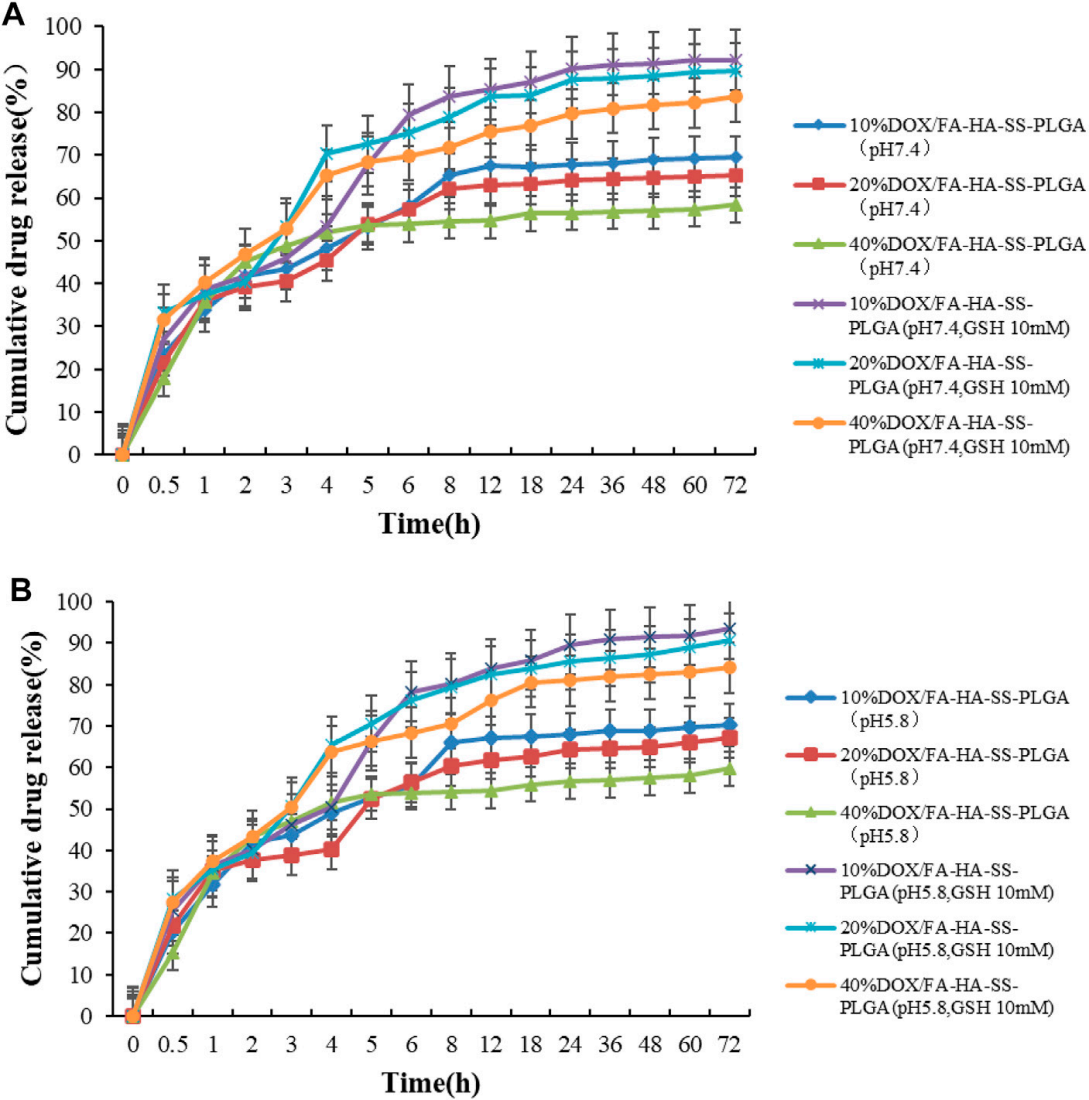
*In vitro* release curve of DOX. **(A)** The *in vitro* release curve of DOX in PBS buffer at pH 7.4; **(B)** *In vitro* release curve of DOX in pH5.8 PBS buffer. Data are expressed as mean ± SEM (*n* = 3).

The *in vitro* release behavior of drug-loaded micelles was studied in PBS buffer (pH 7.4, pH 5.8). Figure 6 showed *in vitro* release profiles of different drug-carrier ratios in the presence or absence of GSH. Micelles with different drug-loading ratios release faster within 8 h, and then their release rate slowed down to a stationary phase, but the difference was that micelles with different drug-loading ratios have different cumulative release rates under the same conditions. The cumulative release of micelles with drug loading ratios of 10%, 20%, and 40% decreased after 72 h. As the amount of hydrophobic drugs increases, the interaction between the drug and the hydrophobic core increases, resulting in a slight decrease in the cumulative release of the drug. In the buffer with a high concentration of GSH, the cumulative drug release was high, and the pH value of the buffer did not change at this point. This indicates that the disulfide bond in micelles was broken, and more DOX was released under the action of a high concentration of GSH, which can enhance the antitumor effect, which was in line with our design expectations. In addition, the PLGA-based micelles were released rapidly within 5–10 h because the PLGA raw materials with a high content of hydroxyacetic acid were used. PLGA is composed of lactic acid (LA) and hydroxyacetic acid (GA). LA is hydrophobic, while GA is relatively hydrophilic. In a certain range, increasing the proportion of GA, PLGA hydrophilic enhanced drug release relative enhancement (Zheng et al., 2004).

### Molecular Docking Results of Drug-Loaded Micelles With Target Proteins

**FIGURE 7:**
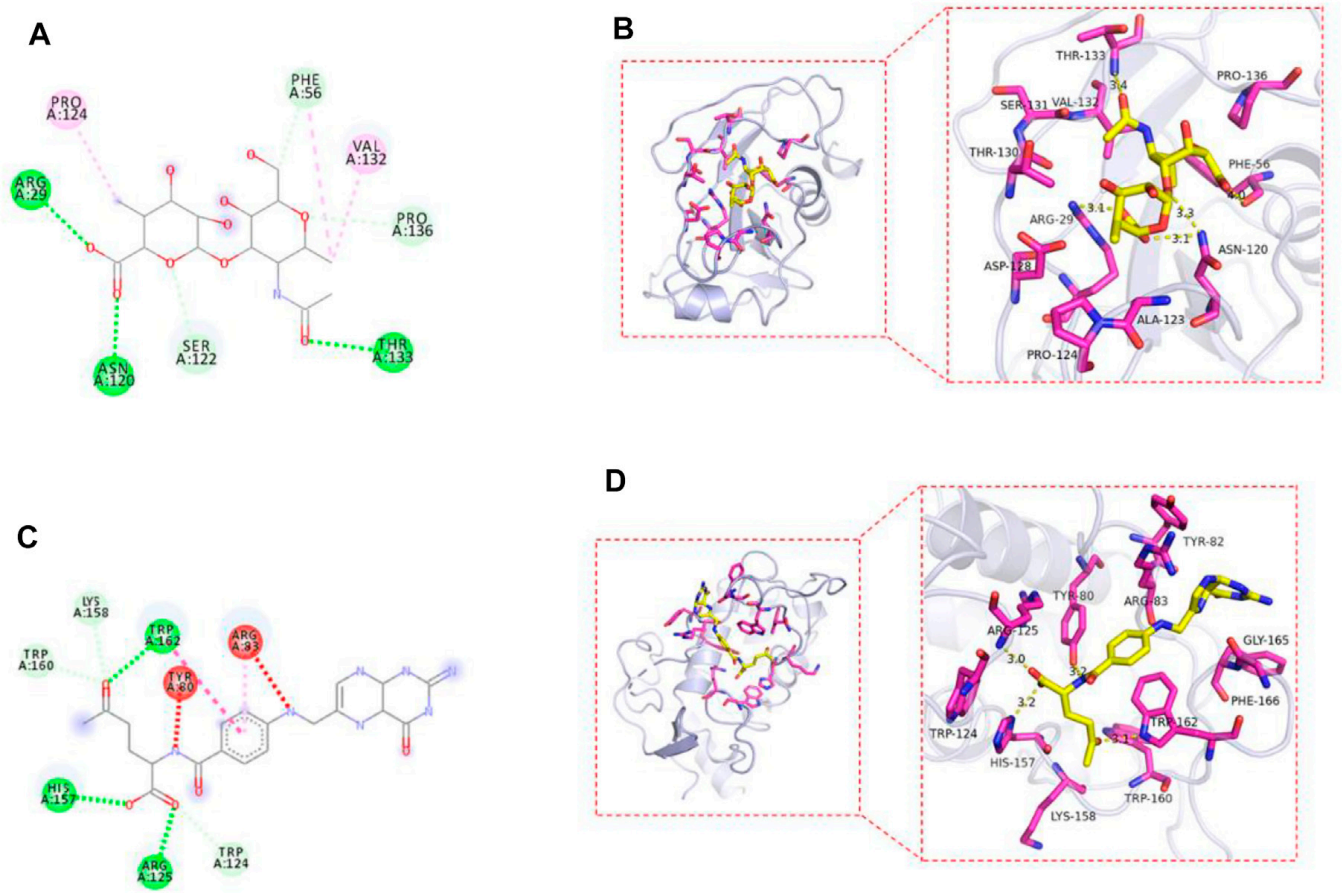
2D plan view of the docking interaction between drug-loaded micelles and CD44 **(A)**; FOLR1 **(C)**. 3D plan view of the docking interaction between drug-loaded micelles and CD44 **(B)**; FOLR1 **(D)**.

The AutoDock Vina1.1.2 software was used to study the molecular docking of the final product. The crystal structures of CD44 protein and FOLR1 protein were obtained from the PDB protein database. The binding model was analyzed by MOE through the prepared ligands and receptors. The 2D and 3D molecular docking diagrams are shown in Figure 7. The binding energy between drug-loaded micelles and CD44 protein was −7.5 kcal/mol, and that between drug-loaded micelles and FOLR1 protein was −7.3 kcal/mol, which proved that drug-loaded micelles had good binding with target proteins, mainly through hydrogen bonding and hydrophobic interaction with target proteins.

### Biocompatibility Analysis

**FIGURE 8:**
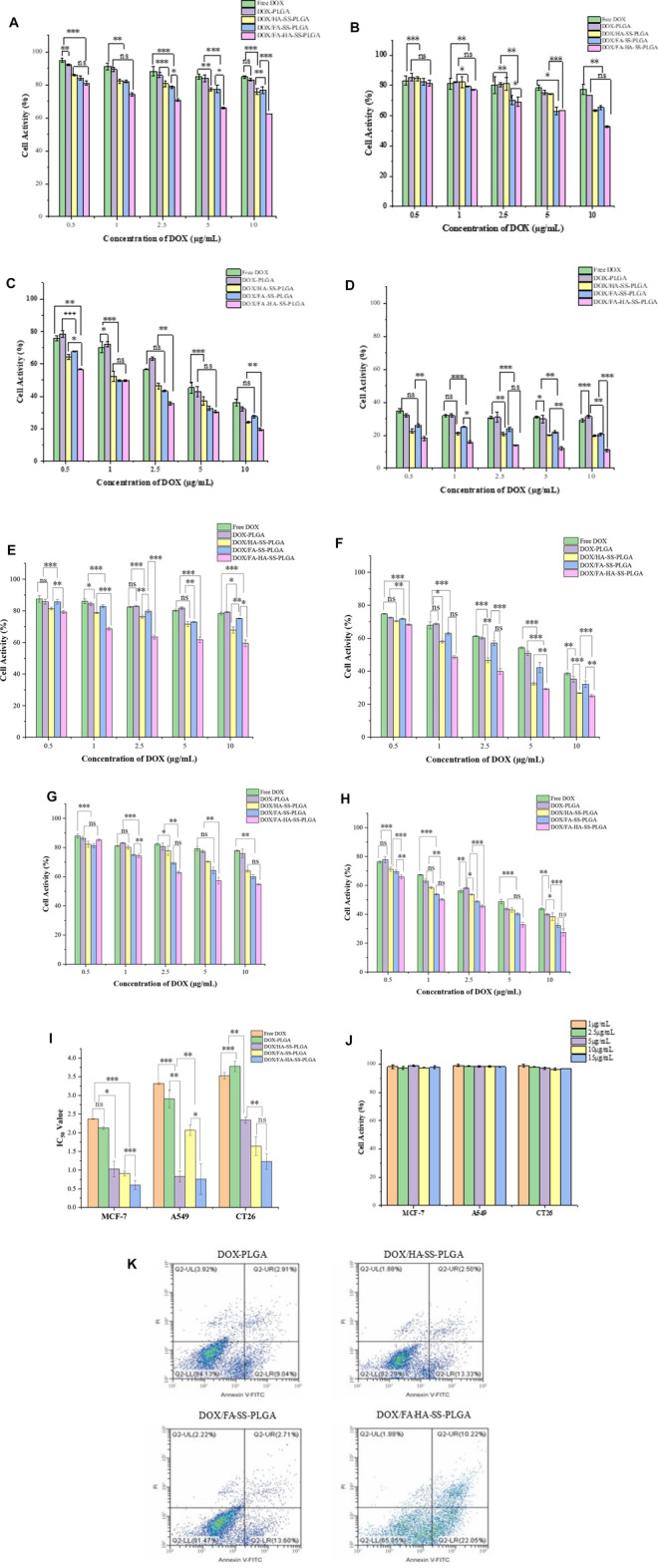
Tumor cell activity. **(A)** Human breast cancer cells (MCF-7) cells were cultured for 12 h. **(B)** MCF-7 cells were cultured for 24 h. **(C)** MCF-7 cells were cultured for 48 h. **(D)** MCF-7 cells were cultured for 72 h. **(E)** A549 cells were cultured for 24 h. **(F)** A549 cells were cultured for 48 h. **(G)** Cell culture of CT26 for 24 h. **(H)** CT26 cell culture for 48 h. **(I)** IC_50_ values of free DOX and different drug-loaded micelles on MCF-7, A549, and CT26 cells cultured for 48 h. The data were expressed as mean ± SEM (*n* = 3). **(J)** Effects of blank micelles at different concentrations on cell viability after 48 h of MCF-7 cell culture. **(K)** Apoptosis of MCF-7 cells treated for 12 h with DOX-PLGA, DOX/HA-SS-PLGA, DOX/FA-SS-PLGA, and DOX/FA-HA-SS-PLGA.

HR test is widely used in the biocompatibility evaluation of biomaterials. HR represents the degree of hemolysis and intracellular hemoglobin dissociation when micelles contact with red blood cells (RBC). The greater the value of HR, the greater the degree of RBC damage. When HR (%) <5%, the micelles showed good blood compatibility. As shown in Table 2, the HR values of all micelles were lower than 5%. The results showed that all micelles had little effect on RBC, indicating that micelles had excellent antihemolytic properties. In addition, the cell viability of MCF-7, A549, and CT26 cells treated with blank micelles was higher than 95% in the concentration range of 1–15 μg/ml (Figure 8). All these proved that the carrier material is a biocompatible, safe, and nontoxic nanocarrier material.

**TABLE 2:** Hemolysis rate of different micelles (*n* = 3, $\bar{x}$ ± SEM).

Type of samples	Absorbance (OD) value	Hemolysis rate (%)
FA-HA-SS-PLGA	0.049 ± 0.003	2.8 ± 0.5
10% DOX/FA-HA-SS-PLGA	0.051 ± 0.005	3.0 ± 0.4
20% DOX/FA-HA-SS-PLGA	0.043 ± 0.003	2.1 ± 0.1
40% DOX/FA-HA-SS-PLGA	0.057 ± 0.007	3.7 ± 0.5
Negative control	0.024 ± 0.003	−
Positive control	0.912 ± 0.007	−

### *In vitro* Evaluation of Cytotoxicity

MCF-7 are cell lines with abundant folate and CD44 receptor expression. Relatively, A549 are cell lines with abundant expression of CD44 receptor and deficient expression of folate receptor, while CT26 are cell lines with abundant expression of folate receptor and deficient expression of CD44 receptor. The cytotoxicity of drug-loaded micelles, blank micelles, and free DOX on MCF-7 cells, A549 cells, and CT26 cells was determined by the MTT method to preliminarily evaluate the medicinal value of micelles. For these three cells, the cell viability was inversely proportional to the concentration of DOX under the same culture conditions. With the increase in culture time, the corresponding cell viability decreased, which was not obvious at a low concentration of DOX, but changed significantly at a high concentration. Micelles with different drug-loading ratios at the same DOX concentration had little difference in cell viability after the same treatment time. For MCF-7 cells, at the highest DOX concentration (10 μg/ml), the cell viability of each group was 36.21% ± 2.57%, 32.23% ± 2.97%, 24.23% ± 3.32%, 27.53% ± 4.02%, and 19.67% ± 3.18% after 48 h of treatment, respectively. DOX/FA-HA-SS-PLGA drug-loaded micelles had the strongest ability to kill tumor cells, showing higher cytotoxicity. This indicated that the internalization of DOX/FA-HA-SS-PLGA was promoted by CD44 receptor and folate receptor-mediated endocytosis, and the antitumor effect of DOX was increased. On the contrary, in folate receptor-deficient A549 cells and CD44 receptor-deficient CT26 cells, decreased cellular uptake reduced the efficacy of DOX. These results showed that drug-loaded micelles containing FA- and HA-targeting ligands had stronger ability to kill tumor cells.

In addition, the antitumor effect of DOX was quantified by half of the maximum inhibitory concentration (IC_50_, 50% cell killing concentration). As shown in Figure 8I, DOX/FA-HA-SS-PLGA micelles increased the antitumor effect of DOX. Compared with MCF-7 cells, the IC_50_ values of DOX/FA-HA-SS-PLGA micelles for A549 cells and CT26 cells were decreased. Due to the high expression levels of CD44 receptors and FRs in most tumor cells, DOX/FA-HA-SS-PLGA drug-loaded micelles can enhance the antitumor effect of DOX and reduce its toxic and side effects on normal cells lacking CD44 receptors and FRs.

The apoptosis of MCF-7 cells induced by different DOX preparations is shown in Figure 8K. DOX/HA-SS-PLGA, DOX/FA-SS-PLGA, and DOX/FA-HA-SS-PLGA produced significantly stronger apoptosis-inducing ability than the DOX-PLGA group. Moreover, a total apoptotic rate (early apoptosis cells plus late apoptotic cells) of MCF-7 cells incubated with DOX/FA-HA-SS-PLGA micelles was 32.27%, which was much higher than the 15.83% of DOX/HA-SS-PLGA micelles and 16.31% of DOX/FA-SS-PLGA micelles. These results indicated that DOX/FA-HA-SS-PLGA drug-loaded micelles would effectively promote the apoptosis of tumor cells and improve the antitumor efficacy of DOX.

### Cellular Uptake

**FIGURE 9:**
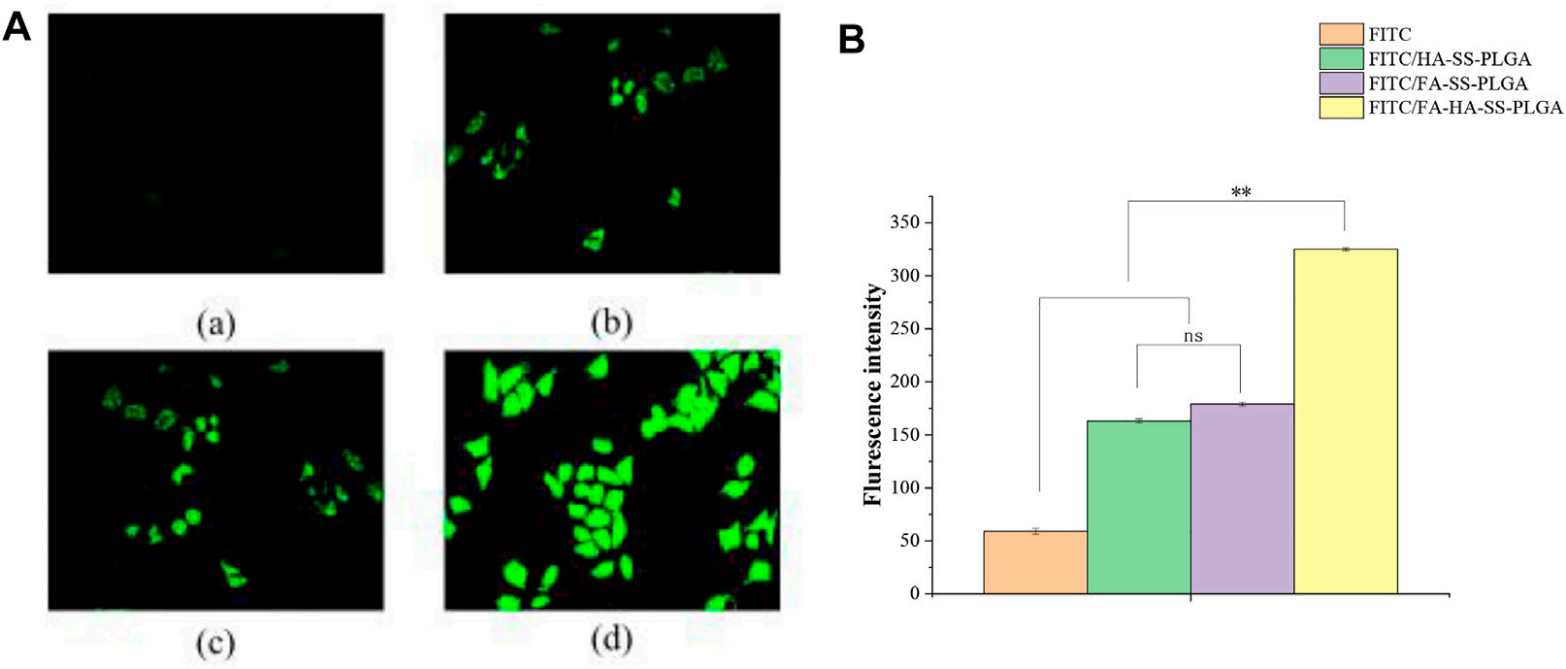
MCF-7 cell uptake study. **(A)** Uptake of free fluorescein isothiocyanate (FITC) (a), FITC/HA-SS-PLGA (b), FITC/FA-SS-PLGA (c), and FITC/FA-HA-SS-PLGA (d) in MCF-7 cells was observed by fluorescence inverted microscope (scale: 50 μm). **(B)** The average fluorescence intensity of each administration group in MCF-7 cells was detected by flow cytometry. Data were expressed as mean ± SEM (*n* = 3).

As shown in Figure 9A, group (d) showed the strongest green fluorescence signal. Figure 9B shows that the mean fluorescence intensity of FITC-loaded micelles in MCF-7 cells was significantly stronger than that of the FITC solution, which may be due to the enhanced endocytosis mediated by specific ligand–receptor interactions. In addition, after 2 h of incubation, the MFI of cells incubated with FITC/FA-HA-SS-PLGA micelles was 1.99- and 1.82-fold of those incubated with FITC/HA-SS-PLGA micelles and FITC/FA-SS-PLGA micelles, suggesting the high intracellular internalization capacity of FITC/FA-HA-SS-PLGA.

### Evaluation of Antitumor Activity and Systemic Toxicity *in vivo*

**FIGURE 10:**
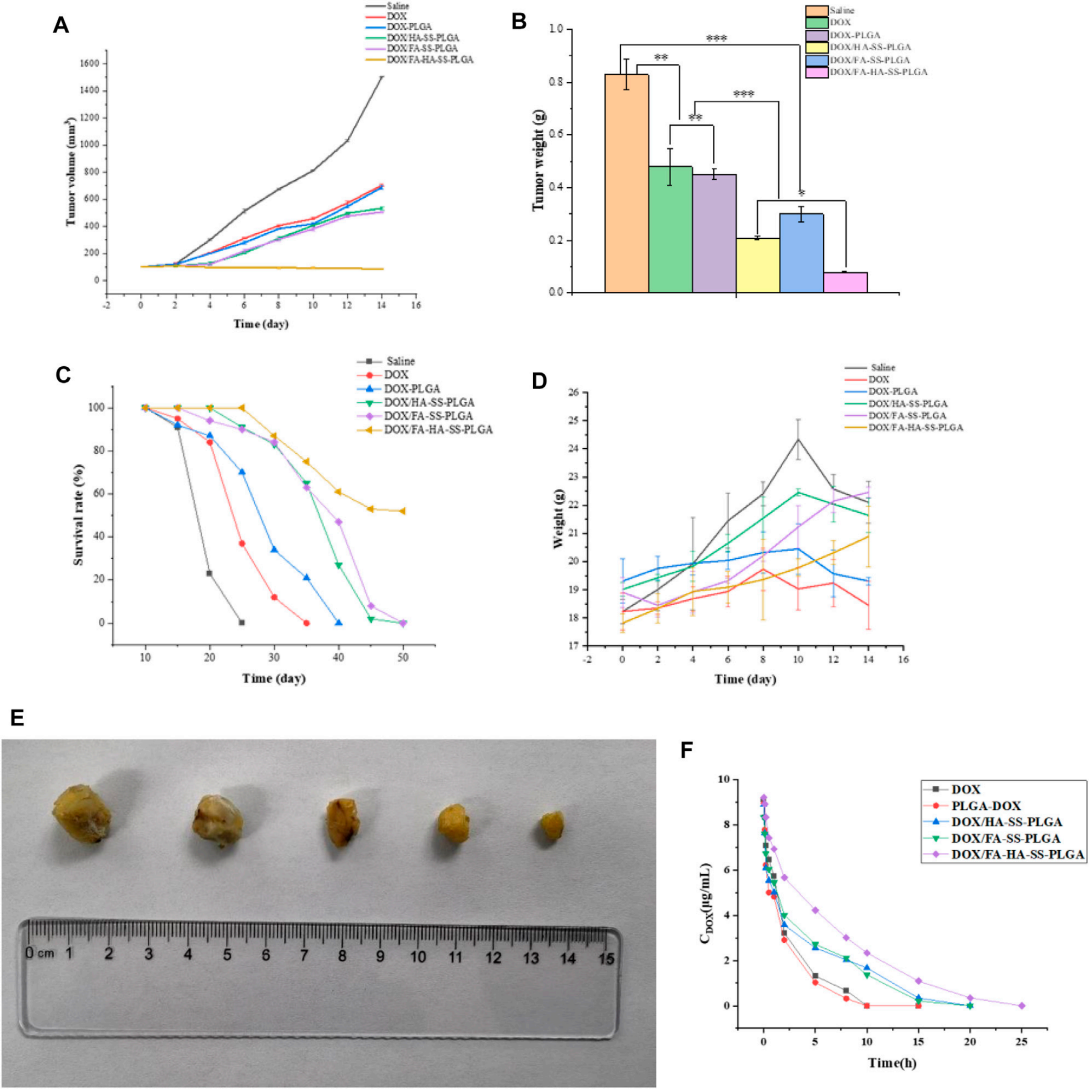
**(A)** The tumor growth volume after treatment with saline, DOX, DOX-PLGA, DOX/HA-SS-PLGA, DOX/FA-SS-PLGA, and DOX/FA-HA-SS-PLGA. **(B)** Tumor weight after treatment with saline, DOX, DOX-PLGA, DOX/HA-SS-PLGA, DOX/FA-SS-PLGA, and DOX/FA-HA-SS-PLGA (*p < 0.05; **p < 0.01; ***p < 0.001). **(C)** Survival rate of mice after treatment with saline, DOX, DOX-PLGA, DOX/HA-SS-PLGA, DOX/FA-SS-PLGA, and DOX/FA-HA-SS-PLGA. **(D)** Changes in body weight of nude mice during experiment. **(E)** Tumor morphology after experiment. **(F)** Pharmacokinetics of various preparations in mice. Data were expressed as mean ± SEM (*n* = 5).

MCF-7 are cell lines with abundant expression of FRs and CD44 receptors. Therefore, MCF-7 cells were used to establish tumor-bearing animal models to study the antitumor performance of drug-loaded micelles *in vivo*. As shown in Figures 10A, B, after the treatment of tumor-bearing mice with saline, DOX, DOX-PLGA, DOX/HA-SS-PLGA, DOX/HA-SS-PLGA, and DOX/FA-HA-SS-PLGA, the group injected with DOX/FA-HA-SS-PLGA showed the strongest tumor inhibitory effect. After 14 consecutive days of injection of DOX/FA-HA-SS-PLGA, the tumor volume was the smallest. Figure 10C showed that the group injected with DOX/FA-HA-SS-PLGA had the highest survival rate, and the average survival time of mice was significantly increased. In addition, the IR was calculated according to tumor weight results (Table 3). The IR value of DOX/FA-HA-SS-PLGA was 2.11 and 1.19 times higher than that of DOX and DOX/HA-SS-PLGA, respectively. These results further indicated that DOX/FA-HA-SS-PLGA had the most effective antitumor effect. These dual-targeted reduction-sensitive drug-loaded micelles had better therapeutic effect on tumors and reduced the side effects of traditional chemotherapy drugs. Finally, the pharmacokinetics of drug-loaded micelles was studied. The *in vivo* elimination half-life of DOX/FA-HA-SS-PLGA was 6.03 h, which was 4.82 and 4.60 times that of DOX and DOX-PLGA, respectively, indicating that this drug-loaded micelles could prolong the action time of DOX.

**TABLE 3:** Inhibitory effects of different preparations on tumor weight and growth in tumor-bearing mice (*n* = 5, $\bar{x}$ ± SEM).

Group	Tumor weight (day 15)	Inhibitory rate (%)
Saline	0.83 ± 0.07	−
DOX	0.48 ± 0.03	42.17
DOX-PLGA	0.56 ± 0.06	41.03
DOX/HA-SS-PLGA	0.21 ± 0.01	74.70
DOX/FA-SS-PLGA	0.35 ± 0.06	78.57
DOX/FA-HA-SS-PLGA	0.09 ± 0.03	89.16

### Results of Doxorubicin Hydrochloride/Folic Acid-Hyaluronic Acid-SS-Poly (Lactic-co-Glycolic Acid) Drug-Loaded Micelle Stability Test

**FIGURE 11:**
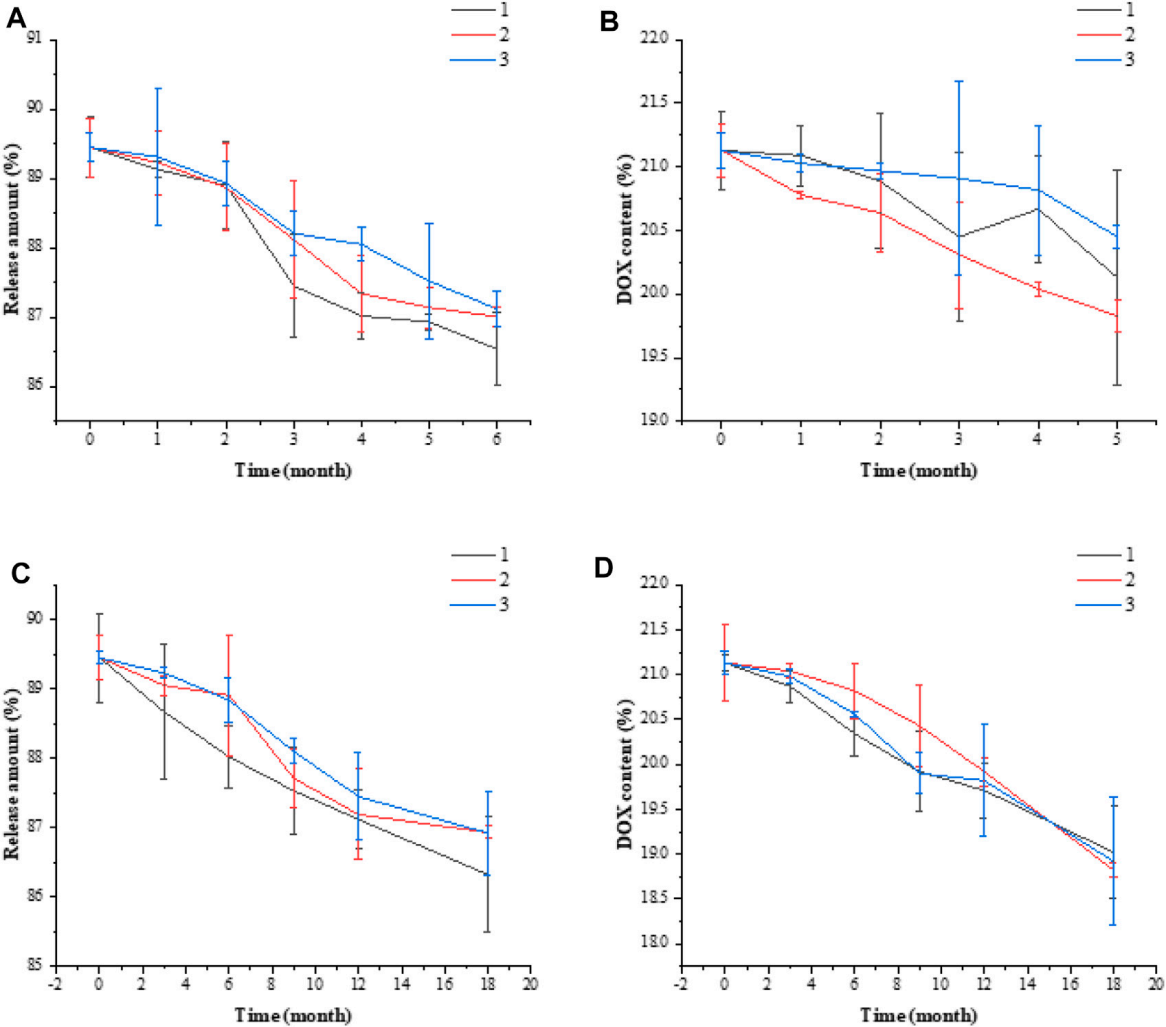
Accelerated stability test release amount **(A)**, DOX content **(B)**. Long-term stability test release amount **(C)**, DOX content **(D)**. Data are expressed as mean ± SEM (*n* = 3).

The experimental results of the influencing factors are shown in Supplementary Table S4. The experimental results showed that the prepared DOX/FA-HA-SS-PLGA drug delivery system should be stored in a closed and dark place. The results of accelerated stability test and long-term stability test are shown in Figure 11. In these two experiments, there was no significant difference in the release amount and DOX content of the DOX/FA-HA-SS-PLGA administration system at different time points, indicating that the stability of DOX/FA-HA-SS-PLGA administration system was good.

## Conclusion

In summary, a novel amphiphilic block micelle DOX/FA-HA-SS-PLGA with dual receptor targeting and reduction sensitivity was prepared, which selectively delivered antitumor drug DOX to kill tumor cells. The preparation scheme was optimized by response surface methodology with drug loading rate and entrapment efficiency as evaluation indexes. The results showed that the preparation process was the best when the drug loading ratio was 30%, the stirring speed was 610 r/min, and the stirring time was 4.2 h. The experimental verification showed that the drug loading rate of the drug loading system was 21.13% ± 1.21%, and the entrapment efficiency was 83.25% ± 0.45%. Its critical micelle concentration was 23.31 ± 1.91 μg/ml, which was verified by stability experiment and had good stability. As expected, DOX can be released from DOX/FA-HA-SS-PLGA micelles stimulated by GSH *in vitro* release studies. In an *in vitro* cytology study, DOX/FA-HA-SS-PLGA micelles enhanced the therapeutic effect of DOX by precise release mediated by double receptors. Compared with FR-deficient A549 cells and CD44 receptor-deficient CT26 cells, micelles showed superior cytotoxicity to MCF-7 cells. Moreover, DOX/FA-HA-SS-PLGA micelles showed significant antitumor effect *in vivo*, with lower side effects and higher survival rate of mice.

Although this micelle can accurately target tumor cells, its permeability in tumor tissues was poor, and its distribution in pathological tissues was uneven after vascular extravasation, which was another obstacle for chemotherapeutic drugs in antitumor efficacy. DOX/FA-HA-SS-PLGA micelles still have shortcomings in solving low permeability, but the progress in improving the antitumor effect of DOX cannot be ignored by experiments.

In short, this dual-receptor targeted reduction-sensitive micelle has great application prospects in tumor treatment.

## Data Availability

The original contributions presented in the study are included in the article/Supplementary Material. Further inquiries can be directed to the corresponding author.
