# Supplementary material for: CD44/Folate Dual Targeting Receptor Reductive Response PLGA-Based Micelles for Cancer Therapy
Source: Front Pharmacol. 2022 Mar 10;13:829590. doi: 10.3389/fphar.2022.829590 (PMC8960309; doi:10.3389/fphar.2022.829590)
Supplement: Supplementary file 1 [file DataSheet1.docx]

Supplementary Material

**Supplementary Table 1.** results of variance analysis of regression equation of drug loading rate

| **Source** | **Sum of squares** | **Df** | **Square** | **F-value** | **Prob>F** |
| --- | --- | --- | --- | --- | --- |
| Model | 64.81 | 9 | 7.2 | 53.94 | < 0.0001 |
| A | 1.81 | 1 | 1.81 | 13.52 | 0.0079 |
| B | 0.031 | 1 | 0.031 | 0.23 | 0.6433 |
| C | 0.011 | 1 | 0.011 | 0.084 | 0.78 |
| AB | 0.14 | 1 | 0.14 | 0.019 | 0.895 |
| AC | 0.20 | 1 | 0.20 | 0.019 | 0.895 |
| BC | 0.16 | 1 | 0.16 | 1.2 | 0.3099 |
| A^2^ | 42.98 | 1 | 42.98 | 321.96 | < 0.0001 |
| B^2^ | 6.79 | 1 | 6.79 | 50.87 | 0.0002 |
| C^2^ | 7.9 | 1 | 7.9 | 59.2 | 0.0001 |
| Residual | 0.93 | 7 | 0.13 |  |  |
| Lack of Fit | 0.68 | 3 | 0.23 | 3.61 | 0.1234 |
| Pure Error | 0.25 | 4 | 0.063 |  |  |
| Cor Total | 65.75 | 16 |  |  |  |

**Supplementary Table 2.** Results of variance analysis of encapsulation efficiency regression equation.

| **Source** | **Sum of squares** | **Df** | **Square** | **F-value** | **Prob>F** |
| --- | --- | --- | --- | --- | --- |
| Model | 70.69 | 9 | 7.85 | 5.55 | 0.0171 |
| A | 4.5 | 1 | 4.5 | 3.18 | 0.1177 |
| B | 0.36 | 1 | 0.36 | 0.26 | 0.6289 |
| C | 7.8 | 1 | 7.8 | 5.51 | 0.0512 |
| AB | 0.3 | 1 | 0.3 | 0.21 | 0.6579 |
| AC | 0.023 | 1 | 0.023 | 0.016 | 0.9032 |
| BC | 0.36 | 1 | 0.36 | 0.25 | 0.6295 |
| A^2^ | 13.87 | 1 | 13.87 | 9.8 | 0.0166 |
| B^2^ | 7.56 | 1 | 7.56 | 5.34 | 0.0541 |
| C^2^ | 30.47 | 1 | 30.47 | 21.53 | 0.0024 |
| Residual | 9.91 | 7 | 1.42 |  |  |
| Lack of Fit | 6.64 | 3 | 2.21 | 2.71 | 0.18 |
| Pure Error | 3.27 | 4 | 0.82 |  |  |
| Cor Total | 80.6 | 16 |  |  |  |

**Supplementary Table 3**. Mathematical fitting model and simulation results of drug release in vitro.

| **Theoretical Model of Drug Release** | **Fitting equation** | **Types of preparations** | **GSH or not** | **pH value** | **Fitting degree（R^2^）** |
| --- | --- | --- | --- | --- | --- |
| Zero-order dynamic model | Q_t_=K_o_t | 10%DOX/FA-HA-SS-PLGA  20%DOX/FA-HA-SS-PLGA  40%DOX/FA-HA-SS-PLGA  10%DOX/FA-HA-SS-PLGA  20%DOX/FA-HA-SS-PLGA  40%DOX/FA-HA-SS-PLGA  10%DOX/FA-HA-SS-PLGA  20%DOX/FA-HA-SS-PLGA  40%DOX/FA-HA-SS-PLGA  10%DOX/FA-HA-SS-PLGA  20%DOX/FA-HA-SS-PLGA  40%DOX/FA-HA-SS-PLGA | Without  Without  GSH  GSH | 7.4  5.8  7.4  5.8 | 0.43  0.34  0.41  0.39  0.38  0.42  0.31  0.34  0.29  0.40  0.32  0.41 |
| First order dynamic model | In(Q_∞_-Q_t_)=K_1_t+K | 10%DOX/FA-HA-SS-PLGA  20%DOX/FA-HA-SS-PLGA  40%DOX/FA-HA-SS-PLGA | Without | 7.4 | 0.96  0.95  0.93 |
|  |  | 10%DOX/FA-HA-SS-PLGA  20%DOX/FA-HA-SS-PLGA  40%DOX/FA-HA-SS-PLGA | Without | 5.8 | 0.91  0.98  0.95 |
|  |  | 10%DOX/FA-HA-SS-PLGA | GSH | 7.4 | 0.97 |
|  |  | 20%DOX/FA-HA-SS-PLGA  40%DOX/FA-HA-SS-PLGA  10%DOX/FA-HA-SS-PLGA  20%DOX/FA-HA-SS-PLGA  40%DOX/FA-HA-SS-PLGA | GSH | 5.8 | 0.92  0.94  0.97  0.96  0.90 |

**Supplementary Table 4.** Test results of influencing factors（n=3, $\bar{\boldsymbol{x}}$±SEM）

| **Condition** | **Time/d** | **Release amount /%** | **DOX content /%** |
| --- | --- | --- | --- |
| - | 0 | 89.45±3.56 | 21.13±1.21 |
| High temperature | 5 | 75.39±2.12 | 18.76±1.73 |
|  | 10 | 63.52±3.12 | 12.65±0.07 |
| High humidity | 5 | 76.21±3.01 | 9.56±0.68 |
|  | 10 | 65.73±1.54 | 8.28±0.75 |
| Strong light | 5 | 78.88±1.52 | 16.02±0.43 |
|  | 10 | 69.25±1.64 | 10.92±0.76 |
